# Quantitative ^1^H and ^13^C NMR and Chemometric Assessment of ^13^C NMR Data: Application to Anabolic Steroid Formulations

**DOI:** 10.3390/molecules30092060

**Published:** 2025-05-06

**Authors:** Stéphane Balayssac, Gaëtan Assemat, Saïda Danoun, Myriam Malet-Martino, Véronique Gilard

**Affiliations:** 1 Laboratoire Softmat, CNRS UMR 5623, Université de Toulouse, F-31062 Toulouse, France; 2 Laboratoire SPCMIB, CNRS UMR 5068, Université de Toulouse, F-31062 Toulouse, Francesaadia.danoun@univ-tlse3.fr (S.D.); marie-catherine.malet-martino@univ-tlse3.fr (M.M.-M.)

**Keywords:** NMR, chemometrics, steroid, substandard drug

## Abstract

This study investigates the potential of ^1^H and ^13^C NMR for the characterization and classification of anabolic androgenic steroids (AASs) in various formulations. First, twenty AAS formulations, including tablets, capsules, and injectable solutions, were analyzed using ^1^H NMR for the qualitative identification and quantification of active compounds. The results revealed discrepancies between the labeled and detected substances in several samples, highlighting issues related to product mislabeling and potential health risks. Then, twelve oil-based injectable formulations were examined using ^13^C NMR, demonstrating its effectiveness in differentiating and quantifying closely related steroid structures that cannot be discriminated with ^1^H NMR. A chemometric approach from ^13^C NMR data, based on a principal component analysis (PCA) and hierarchical cluster analysis (HCA), enabled the classification of samples and the identification of key active ingredients.

## 1. Introduction

Nuclear magnetic resonance (NMR) is a powerful analytical tool that has proven highly effective in detecting poor-quality or substandard products. It has been widely applied in various fields, including food authentication, pharmaceuticals, and forensic science, particularly for analyzing adulterated dietary supplements and counterfeit medicines [1,2,3,4,5]. This study aims to explore the potential of NMR in the analysis of androgenic anabolic steroid (AAS) formulations.

Physical activity and sports are an integral part of daily life for many individuals, not only professional but also recreational athletes, particularly in high-income countries. In the pursuit of enhanced performance, some individuals turn to unauthorized substances, including AASs, which are commonly used to promote muscle growth and improve strength [6]. While these compounds are strictly regulated and often prohibited, they remain accessible through illicit and Internet markets in various forms, including tablets, capsules, and injectable solutions. The World Anti-Doping Agency publishes an annual list of banned substances in sport [7], which includes compounds prohibited either at all times or only in competition. Among these, AAS represent one of the most frequently encountered classes. Despite regulations, the manufacturing and distribution of anabolic steroid formulations remain a significant concern, with products often misrepresented, adulterated, or containing undeclared active substances. The forensic analysis of AAS formulations is essential in combatting their illicit distribution and ensuring compliance with legal regulations. A review by Berneira et al. provides a comprehensive overview of the extraction methods and analytical techniques used in the detection of AASs in seized formulations, summarizing the advances from 1979 to 2021 [8]. Conventional screening methods rely on chromatographic techniques such as gas chromatography (GC) or liquid chromatography (LC) coupled with UV detection (DAD) or mass spectrometry (MS) [9,10,11]. The detection and characterization of anabolic steroids in injectable formulations pose a major analytical challenge. In that case, the most common techniques include LC-UV and LC-MS [12,13], as well as GC-MS [9,14].

In 2018, proton nuclear magnetic resonance (^1^H NMR) was described as an efficient tool for detecting anabolic steroid adulterations in seized drug samples [15,16]. Building on this, our study goes beyond ^1^H NMR by also investigating **^13^C NMR,** combined with chemometric data analysis, to enhance the characterization and classification capabilities. Indeed, the combination of NMR spectroscopy with chemometric techniques is emerging as a powerful trend in analytical science, enhancing the quality assessment of complex matrices across various fields, including pharmaceuticals and food authentication [17,18].

This study, based on samples seized by French law enforcement authorities, was conducted in two stages according to the samples received. First, 20 AAS formulations were analyzed by ^1^H NMR as a quality control method to verify the compliance of the samples in terms of claimed active compounds and dosage. A few months later, 12 additional AAS formulations, all packaged in oily matrices, were analyzed both qualitatively and quantitatively. Since the claimed composition of some of these samples was more complex, ^1^H NMR proved to be a limited method. We, therefore, turned to ^13^C NMR combined with chemometrics in order to develop an analytical strategy for sample classification and the identification of active compounds.

## 2. Results

### 2.1. ^1^H NMR for the Assessment of 20 AAS Formulations

In the first step, ^1^H NMR was used as a profiling method. The spectra were obtained after an easy sample preparation as described in the experimental section. The presence of active compound(s) in each sample was clearly revealed. Their identification was carried out by comparing the ^1^H NMR spectra of the samples with those of standard compounds when available. In cases where standards were not available, two-dimensional NMR experiments were performed to aid in the structural elucidation. The cross-validation of the active structure identification was carried out by high-resolution tandem mass spectrometry (MS/MS) in the direct infusion mode. All MS data are reported as Supplementary Materials in Table S1.

**Figure 1:**
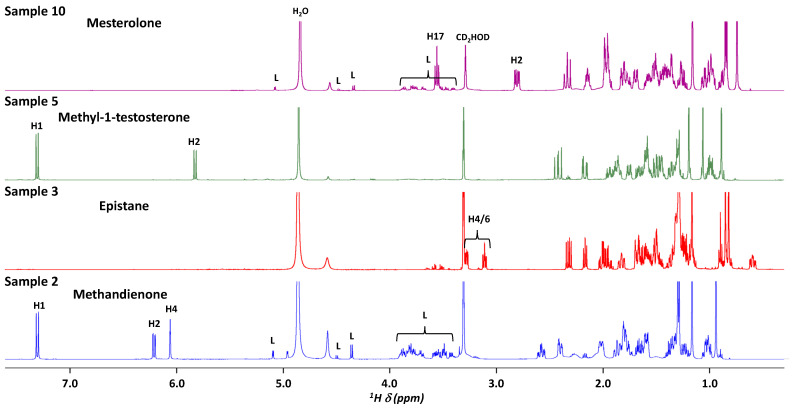
^1^H NMR spectra of the four formulations **2**, **3**, **5**, and **10**. Spectra were recorded at 500 MHz in methanol-d4. L: Lactose. The proton numbering refers to Figure A1 (Appendix A.1).

Table 1 summarizes the data for all samples, highlighting the claimed and detected products. Figure 1 shows typical ^1^H NMR spectra recorded for a few samples (**2**, **3**, **5,** and **10**) in methanol-d4. Lactose, used as an excipient, is detected in formulations **2** and **10**, which are sold in tablet forms. Other main signals detected correspond to the claimed active compound with a complex spectral pattern of aliphatic protons of the steroid skeleton in the area 0.9–2.7 ppm. The characteristic signals of unshielded protons were H17 at 3.56 ppm (triplet (t)) and H2 at 2.79 ppm (doublet of doublet (dd) (14.7, 6.0)) for mesterolone (formulation **10**), and H4/H6 episulfide protons at 3.28 and 3.11 ppm (multiplets (m)) for epistane (formulation **3**), as well as ethylenic protons for methyl-1-testosterone (H1 at 7.31 ppm, doublet (d) (10.2 Hz), H2 at 5.83 ppm, doublet of doublet (dd) (10.2, 0.7 Hz)) (formulation **5**), and methandienone (H1 at 7.31 ppm, d (10.0 Hz), H2 at 6.21 ppm, dd (10.0, 1.9 Hz), and H4 at 6.06 ppm, apparent triplet (apt) (1.9 Hz)) (formulation **2**).

**Figure 2:**
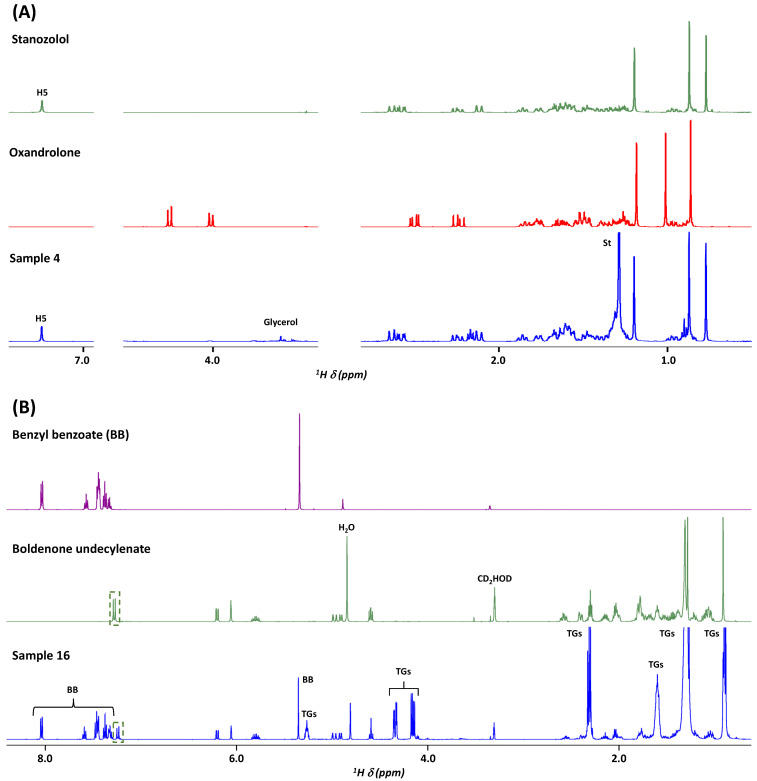
^1^H NMR spectra recorded at 500 MHz in methanol-d4: (**A**) spectrum of formulation **4** overlaid with those of the claimed standard (oxandrolone) and the detected standard (stanozolol); and (**B**) spectrum of formulation **16** overlaid with those of the claimed standard (boldenone undecylenate) and one excipient (benzyl benzoate). St: Stearate, BB: Benzyl benzoate, TGs: Triglycerides.

Figure 2A presents the case of sample **4**, a tablet formulation claimed to contain oxandrolone. However, the spectral overlay shows that the characteristic signals of oxandrolone, such as the AB system for H3 protons (4.26 (part A) and 4.01 (part B) ppm in Figure 2A), are absent. Instead, the ^1^H NMR profile matches that of stanozolol, another AAS, such as, for instance, the H5 aromatic proton of the pyrazole ring at 7.24 ppm. Additional signals come from excipients such as glycerol or stearate, with magnesium stearate being a common excipient in tablets.

The spectrum shown in Figure 2B corresponds to the injectable formulation **16**. In this sample, the matrix consists of an oily base generating broad signals characteristic of triglycerides, including protons from the glycerol backbone and long-chain fatty acids. The formulation also contains the excipient benzyl benzoate, an adjuvant used to solubilize the active compounds. The expected active compound, boldenone undecylenate, is detected, as indicated by its strong match with the spectrum of the standard compound. From all qualitative results summarized in Table 1, it appears that three samples were incorrect in terms of active compounds, i.e., samples **4** (which contains stanozolol), sample **7** (which contains a mixture of testosterone decanoate and stanozolol), and sample **9** (which contains methyltestosterone).

In the next step, the ^1^H NMR quantification of each active compound was performed using sodium 2,2,3,3-tetradeutero-3-trimethylsilyl-propionate (TSP) as an internal standard. The aim is not to detail the ^1^H NMR quantification procedure, which meets all prerequisites for fully quantitative results, including optimized acquisition and processing parameters [1,15]. Table 2 reports the characteristics of the signals targeted for quantification for each active that were selected to be as isolated as possible to facilitate integration. All quantifications were performed in triplicate.

**Figure 3:**
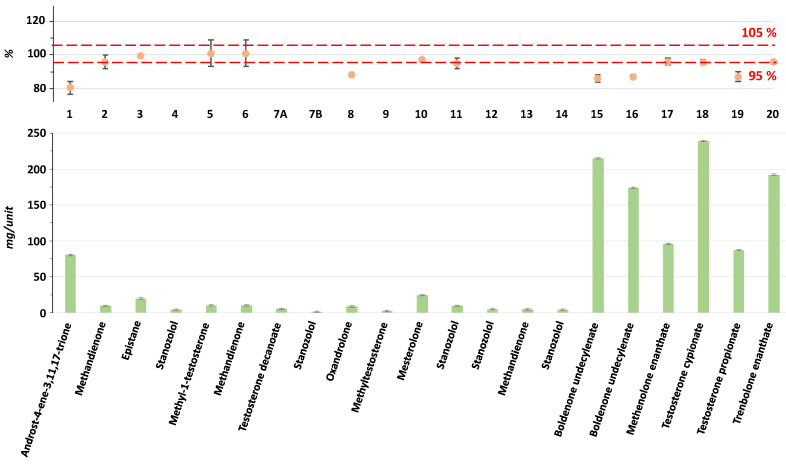
Quantitative analysis of active ingredients detected in the twenty AAS formulations. Concentrations are given in mg per unit (tablet or capsule). For injectable formulations containing 10 mL of solution, the injected dose (unit) is considered to be 1 mL. In the upper part, the percentage differences between the claimed amount and the measured values are reported. The red dotted lines represent the ±5% established limits for active pharmaceutical ingredients.

The results of the quantification are reported in Table S2 and summarized in Figure 3. Compliance for the percentage of actives detected compared to the claimed dosage is not achieved for samples **1** (80 ± 4%), **8** (88 ± 1%), **15** (86 ± 3%), **16** (87 ± 1%), and **19** (87 ± 3%), i.e., for 5 formulations out of 14 (36%). In Figure 3, these five samples appear outside the range of 95–105% (limits established for an active pharmaceutical ingredient by the US FDA) defined by the red lines, while the others remain inside. The detection of inconsistencies in both composition and quantity underscores the necessity of systematic quality control in such products. Moreover, the mislabeling observed in some samples (3 out of 17 formulations; 18%) raises concerns regarding regulatory compliance and potential health risks.

These first results underscore the effectiveness of ^1^H NMR as a rapid and reliable method for screening AAS formulations, both for identifying active compounds and assessing their dosage accuracy. However, the technique has certain limitations. The spectral similarities between different steroids can hinder precise identification, particularly for the structure of the ester moiety. In such cases, it becomes necessary to rely on MS and MS/MS analyses.

### 2.2. Comprehensive NMR and Chemometric Analysis of 12 Oily Injectable AAS Formulations

#### 2.2.1. Qualitative ^1^H and ^13^C NMR Analysis

A second set of 12 oil-based injectable formulations was received for analysis, expected to contain various AASs either alone or in combination (Table 3).

**Figure 4:**
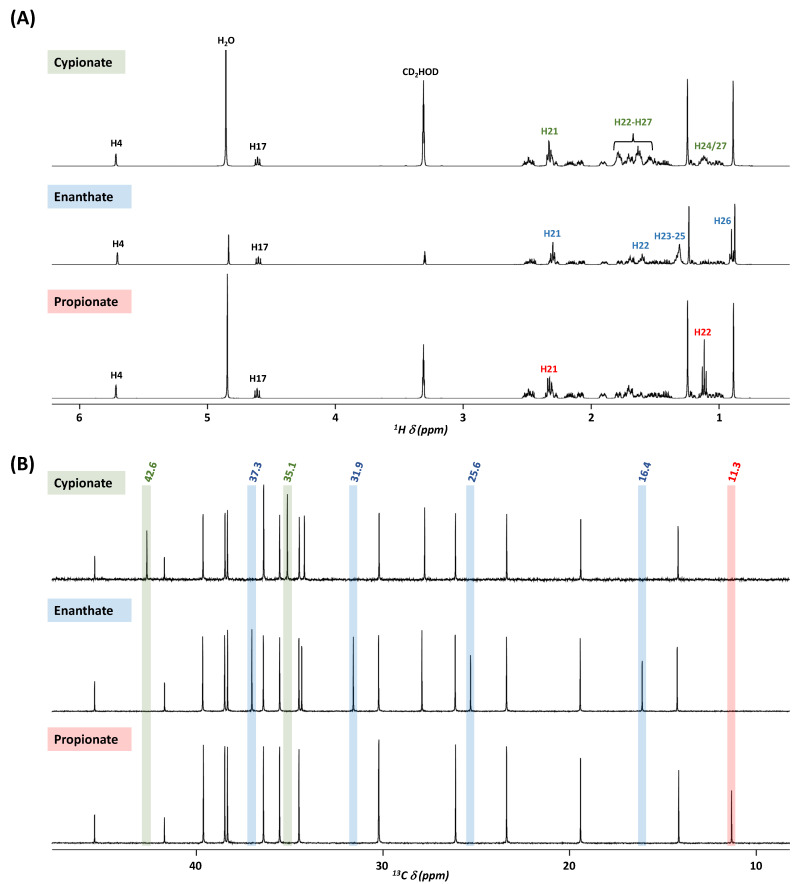
^1^H NMR spectra (**A**) and ^13^C NMR spectra (0–50 ppm region) (**B**) in methanol-d4 of the three standard testosterone esters claimed to be in the **OI-9** formulation. The ester-specific ^13^C NMR signals are highlighted: propionate in red, enanthate in blue, and cypionate in green.

Their ^1^H NMR spectra displayed the characteristic profile of anabolic steroids, along with NMR signals corresponding to benzyl benzoate, and triglycerydes with a glycerol backbone and long-chain fatty acids, as shown in the previous section (Figure 2B). However, unlike the previous formulations **15**–**20** where only one active substance was mentioned, 5 out of the 12 present injectable solutions are expected to contain a combination of three active ingredients. The high similarity between the ^1^H NMR profiles of AASs makes it challenging to identify and quantify these compounds in mixtures. For example, formulation **OI-9** is supposed to contain three testosterone esters (propionate, enanthate, and cypionate). As the protons of the steroid core of testosterone have the same chemical shifts, the NMR signals of the ethylenic proton H4 at 5.70 ppm and of the H17 proton near the ester function at 4.61 ppm can no longer be used for their discrimination. The characteristic chemical shifts of these esters corresponding to the protons H21 and H22 for propionate, H21 to H26 for enanthate, and H21 to H27 for cypionate are located in the 0.85–2.55 ppm region with significantly overlapped signals (Figure 4A).

An alternative NMR approach is to conduct ^13^C experiments, which offer a wider chemical shift ranging from 0 to 200 ppm, minimizing the overlap compared to the proton spectra, and thus providing more detailed information. The nine anabolic steroids listed in the twelve oil-based injectable formulations display distinctive NMR profiles, notably in the 0–50 ppm region of the spectra, despite the large number of NMR signals. As shown in Figure 4B, the spectra of the three standard testosterone esters claimed to be in the **OI-9** formulation are recognizable by their ^13^C NMR profiles, each displaying at least one characteristic signal.

In this comparison, distinct and well-resolved signals were detected for testosterone propionate at 11.3 ppm, for testosterone enanthate at 16.4, 25.6, 31.9, and 37.3 ppm, and for testosterone cypionate at 35.1 and 42.6 ppm. The ^1^H and ^13^C NMR assignments of the three standard testosterone esters are reported in Table A1 (Appendix A.2) and typical correlations within the ester moieties can be visualized in the overlay of the HSQC spectra as shown in Figure S2.

The analysis of the twelve formulations (**OI-1** to **OI-12**) demonstrated that only three testosterone esters (propionate, cypionate, and enanthate) were present, either alone or in combination. As in the first part of the article, the structure identification was confirmed by high-resolution tandem mass spectrometry (MS/MS) in direct infusion mode. All MS data are provided as Supplementary Materials in Table S3. The identification of testosterone esters aligns with those determined by ^13^C NMR, with only trace amounts of other AASs detected by MS in two samples.

#### 2.2.2. Chemometrics

Building on the above findings, we implemented a chemometric approach to analyze the ^13^C NMR spectra of injectable formulations without requiring a prior spectral assignment. The presence of characteristic carbon signals for different testosterone esters provides a basis for this strategy, allowing for an unsupervised statistical analysis of spectral variations. This method aims to reveal the natural clustering among samples and makes possible the identification of active ingredients.

**Figure 5:**
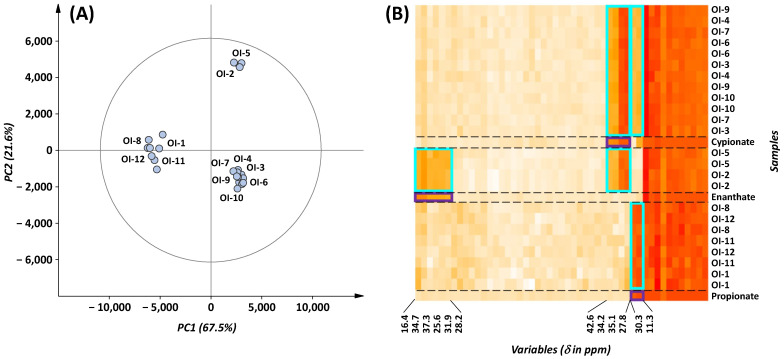
Chemometric analysis of ^13^C NMR dataset. (**A**) Score plot of a PCA built from the twelve oil-based injectable formulations analyzed in duplicate (*n* = 24). (**B**) Heatmap of the injectable formulations and the three suspected testosterone esters (propionate, enanthate, and cypionate). Colored rectangles highlight the discriminating variables (chemical shifts), with purple frames indicating those distinguishing the testosterone esters and the cyan blue frames representing those identified in the samples.

After the pre-processing and the pre-treatment of the 24 spectra of the **OI-1** to **OI-12** formulations (see Section 4), an unsupervised principal component analysis (PCA) was built, limited to the region of 0–50 ppm. This analysis provided an initial overview of the dataset and revealed clustering trends, as shown in Figure 5A.

Three well-separated clusters appear along the first two components (R^2^X = 0.89) in the PCA score plot. The first cluster included four samples: **OI-1**, **OI-8**, **OI-11**, **OI-12**. The second cluster consisted of two samples: **OI-2** and **OI-5**. The last cluster, comprising six samples, **OI-3**, **OI-4**, **OI-6**, **OI-7**, **OI-9**, and **OI-10**, represents the most significant grouping. Contrary to the information listed on the packaging which indicated different compositions for 11 out of the 12 samples (Table 3), only three characteristic spectral patterns were observed in the PCA analysis.

The ^13^C NMR signals driving the clustering were identified on the PCA loading plot (Figure S1) with two chemical shifts associated with the first cluster, and five associated with both the second and third clusters. Several of those signals had previously been observed (Figure 4B and Figure S2) for the ester moieties of AASs, at 11.3 ppm for propionate, at 16.4, 25.6, 31.9, and 37.3 ppm for enanthate, and at 35.1 and 42.6 ppm for cypionate. Additional signals at 30.3 ppm (propionate); 28.2 and 34.7 ppm (enanthate); and 27.8, 34.2, and 34.6 ppm (cypionate) were highlighted in the PCA loading plot even if their chemical shifts are close to (<0.5 ppm) or partially overlapped with signals from other compounds. Then, to determine which steroid core is present in the formulations, the 0–50 ppm ^13^C NMR data of the samples were compared to those of the nine standards claimed (trenbolone acetate, testosterone enanthate, testosterone cypionate, nandrolone decanoate, boldenone undecylenate, methenolone, drostanolone propionate, testosterone propionate, and trenbolone hexahydrobenzyl carbonate) included in the same dataset, using the Pearson correlation coefficient (Pcc). A Pcc higher than 0.65 was obtained for the 12 formulations, but only for the testosterone moiety. In contrast, no correlation was observed for other steroid patterns, with the following values: <−0.06 for trenbolone, <0.2 for nandrolone, <0.17 for boldenone, <0.15 for methenolone, and <−0.03 for drostanolone. The composition of the twelve injectable formulations, therefore, relies on at least one of the following testosterone esters: propionate, enanthate, and cypionate.

To refine the analysis and determine the presence and the number of active ingredients in the injectable formulations, a further in-depth investigation was necessary. In this context, a hierarchical cluster analysis (HCA), another unsupervised multivariate analysis technique, is particularly useful for exploring patterns and relationships between samples by generating dendrograms and/or heatmaps. The heatmap provides a clear visual representation of the distribution of each NMR signal within the clusters of each formulation. It reorganizes the rows and columns based on the dendrogram grouping similar formulations and chemical shifts closer together. A heatmap of the NMR data, including the 24 ^13^C NMR spectra of the 12 formulations and those of the 3 standard testosterone esters, is presented in Figure 5B. Distinct color patterns corresponding to the testosterone esters (framed in purple) facilitates the identification of these compounds in each formulation (framed in cyan blue). The first group (**OI-1**, **OI-8**, **OI-11**, and **OI-12**) contains only testosterone propionate, the second group (**OI-2** and **OI-5**), a mixture of testosterone cypionate and testosterone enanthate, and the third group (**OI-3**, **OI-4**, **OI-6**, **OI-7**, **OI-9**, and **OI-10**), a combination of testosterone cypionate and testosterone propionate. From the heatmap analysis, no other AAS appears to be present in these formulations.

In conclusion, based on the direct analysis of the ^13^C NMR spectra and their chemometric evaluation, it appears that the claimed composition of the 12 oil-based injectable formulations does not match with the identified compounds: for seven samples, the active ingredients are different from those announced, while, in the remaining five samples, only 1 of 2, 1 of 3, or 2 of 3 listed AASs are actually present. In summary, none of the samples are qualitatively in compliance with the stated composition. We also demonstrated that, even without prior knowledge of NMR signal assignments, a chemometric approach based solely on the ^13^C NMR profiles is a powerful tool for identifying AASs in formulations, provided that the NMR spectra of the suspected pure compounds are available.

2.2.3. ^13^C NMR Quantification

Both the direct and the chemometric analyses showed that the characteristic signals of the three testosterone esters that can be used for their quantification are those of the ester moieties. The absolute quantification was thus performed on one isolated NMR signal without nearby interfering signals, at 11.3 (C22), 16.4 (C26), and 42.6 (C23) ppm for propionate, enanthate, and cypionate, respectively (Table A1 (Appendix A.2)). A chemometric analysis of the ^13^C NMR spectra revealed the characteristic signals of testosterone esters: C21 and C22 for propionate, C21–C26 for enanthate, and C22–C27 for cypionate. The only missing signal on the heatmap was C21 of cypionate, overlapped with the C6 signal at 36.4 ppm (Figure S2).

The NMR quantification requires the complete relaxation of the signals depending on the longitudinal relaxation T_1_. Inversion-recovery experiments were conducted on the **OI-1** and **OI-2** formulations to determine the T_1_ of the relevant NMR signals. The T_1_ values were 4.2, 4.7, and 3.5 s for C22 (propionate), C26 (enanthate), and C23 (cypionate), respectively. The longest T_1_ value was observed for the quantification reference, trioxane, at 13.5 s. To achieve a recovery rate of nearly 99.9%, the experiment time should exceed 22 h for each sample, decreasing to 16 h with a 30° pulse. The addition in the NMR tube of Cr(acac)_3_, a relaxation agent, reduced all the T_1_ values below 3.1 s, allowing the experiment time to be shortened to approximately 4 h. The acquisition time, the ^1^H decoupling, and the number of scans were also optimized to ensure an adequate signal-to-noise ratio (≈30).

The quantification results presented in Table 3 reveal that, for the five formulations that partially match the declared composition (**OI-2**, **OI-4**, **OI-7**, **OI-8**, and **OI-9**), all actives are underdosed, with values ranging from 17% (testosterone cypionate and enanthate in **OI-2**) to 73% (testosterone propionate in **OI-9**) of the stated amounts.

For the four formulations, **OI-1**, **OI-8**, **OI-11**, and **OI-12**, containing only testosterone propionate, quantification was also performed using ^1^H NMR, as described in the first part. The average ratios of the quantified amounts obtained by ^13^C NMR compared to ^1^H NMR were 98.5 ± 3.5%, demonstrating a close agreement and confirming the consistency of ^13^C NMR for the quantification of testosterone esters.

Overall, all the twelve analyzed injectable formulations were of poor quality, with a mismatch between the advertised and actual contents. Interestingly, formulations **OI-3**, **OI-4**, **OI-6**, **OI-7**, **OI-9**, and **OI-10**, all containing a combination of testosterone cypionate and propionate, exhibit very similar dosage levels. This raises the question of whether these products may originate from the same formulation, marketed under different names.

## 3. Discussion

Analytical techniques play a crucial role in ensuring the quality and authenticity of pharmaceutical compounds. In the case of AASs, while these substances are often commercialized outside any legal framework, the presence of undeclared or substituted steroids can lead to unintended physiological effects, highlighting the need for reliable verification methods to detect fraud or inconsistencies [6,19].

In the present study, both qualitative and quantitative results obtained using ^1^H NMR align with those of Ribeiro et al. [16], who also found, through a ^1^H NMR analysis of AAS formulations, that some samples contained unlisted active ingredients, while others had dosages below the stated amount. Similarly, the overall findings from ^1^H and ^13^C NMR corroborate the data presented in the review by Magnolini et al. [20], which highlights the occurrence of substandard AAS formulations.

While ^13^C NMR has long been used for the structural characterization of steroids [21], its application as a screening tool for suspect samples, combined with a chemometric analysis, is entirely novel. However, a similar strategy combining paper spray mass spectrometry (PS-MS) and chemometric models has been successfully applied to seized oily injectable forms of AAS [22]. The chemometric approach presented here, although tested on a limited number of samples, faces limitations in terms of statistical significance and generalizability. This underscores the importance of expanding the dataset in future studies. With a larger sample size, the approach could be scaled up for the development of robust classification models. If a reference library of ^13^C NMR spectra covering a wide range of steroid structures were established, the proposed workflow could enable the identification of steroids in unknown samples, even without in-depth NMR expertise. In the future, integrating machine-learning algorithms [23] such as deep learning or support vector machines, could enhance this approach by uncovering complex patterns of closely related compounds [24]. This could further refine classification models and enhance detection strategies, particularly in the context of counterfeit medicines.

Even though several chromatographic screening methods for AASs in dietary supplements or other formulations already exist [13,14], it remains essential to develop new analytical approaches. A key strength of NMR is its ability to perform quantitative analyses without requiring a specific standard. This study demonstrates that both ^1^H and ^13^C NMR can be effective for this purpose. Obviously, the low sensitivity of NMR, especially ^13^C, can be a limitation. This is particularly pronounced when dealing with complex mixtures or low-concentration components. However, in the context of potential fake formulations, this is not a major concern, as the concentrations of active ingredients (correct or incorrect) in these formulations are generally high, well above the quantification limits. Our findings confirm the relevance of NMR-based quality control in counterfeit drug detection strategies, expanding its applicability to AASs. In particular, the use of ^13^C NMR combined with a chemometric approach provides additional structural insights, further enhancing the detection and classification of these substances. The ability of NMR to detect such inconsistencies highlights its potential role in pharmaceutical quality control, particularly in combating counterfeit or adulterated drugs or dietary supplements. Implementing this methodology in regulatory frameworks, such as those used by anti-doping agencies or drug enforcement authorities, could provide an orthogonal and complementary tool to existing MS-based techniques.

## 4. Materials and Methods

### 4.1. Materials

Samples of supplements and oil-based formulations were obtained from French gendarmerie (law enforcement authorities) seizures.

All chemicals were of analytical grade and were purchased from following suppliers, Sigma-Aldrich (St. Louis, MO, USA), Alfa Aesar (Haverhill, MA, USA), or Acros Organics (Geel, Belgium). The two internal references used, sodium 2,2,3,3-tetradeutero-3-trimethylsilyl-propionate (TSP) and 1,3,5-trioxane, were purchased from Sigma-Aldrich, as was the relaxation agent chromium(III) acetylacetonate (Cr(acac)_3_).

### 4.2. Sample Preparation

#### 4.2.1. Qualitative Analysis

For the qualitative analysis of solid formulations, approximately 100 mg of powder from capsule content or crushed tablet was accurately weighed and mixed with 1 mL of deuterated methanol. The solution was vortexed for 15 s and then subjected to ultrasonic bath sonication for 5 min. Subsequently, the suspension was centrifuged at 3000 rpm for 5 min. A total of 700 μL of the supernatant solution was collected and mixed with TSP, the internal chemical shift reference. The resulting solution was homogenized by vortexing and then transferred into a 5 mm NMR tube (Eurisotop, Cambridge Isotope Laboratories, Saint-Aubin, France). For the qualitative analysis of injectable solutions, 150 mg (approximately 200 μL) were dissolved in 1 mL of deuterated methanol. The solution was vortexed for 15 s and then sonicated for 5 min. The suspension was centrifuged at 3000 rpm for 5 min. A total of 700 μL of the supernatant solution was collected and mixed with TSP (^1^H experiment) or trioxane (^13^C experiment). The resulting solution was homogenized by vortexing and then transferred into a 5 mm NMR tube.

#### 4.2.2. Quantitative Analysis

- Quantitative ^1^H NMR Analysis of Solid Formulations

A variable amount (5 to 50 mg) of powder was accurately weighed and dissolved in 1 mL of methanol-d4, except for sample **5** which was analyzed in DMSO-d6 for optimal solubilization. In each case, the solution was vortexed for 15 s, sonicated for 10 min, and stirred magnetically for 20 min. The obtained solution was centrifuged at 3000 rpm for 5 min. A total of 800 μL of the resulting supernatant was collected, and 30 μL of a 5 mM TSP solution (used as an internal quantification reference) was added. The final solution was vortex-homogenized for 15 s and transferred into an NMR tube.

- Quantitative analysis of injectable formulations

For the quantitative ^1^H NMR analysis of injectable formulations, approximately 50 mg of oil was accurately weighed and extracted with 1 mL of methanol-d4. The extraction procedure involved 15 s of vortexing followed by 10 min of sonication. The resulting solution was centrifuged at 3000 rpm for 5 min. A total of 800 μL of the supernatant was collected, and 100 μL of a 5 mM TSP solution was added. The final solution was vortexed for 15 s and transferred into an NMR tube. The residual solution was extracted again with 1 mL of deuterated methanol following the same procedure as the first extraction. The total product quantity was calculated by accounting for both successive extractions.

For the quantitative ^13^C NMR analysis, approximately 50 mg of an injectable solution, precisely weighed, was mixed with 1 mL of methanol, vortexed for 15 s, sonicated for 10 min, and then centrifuged at 3000 rpm for 5 min. A volume of 800 μL of the supernatant was collected, and the remaining residue was re-extracted with 1 mL of methanol using the same procedure.

Three additional 50 mg oil samples were treated in the same way to ensure a sufficiently high concentration in the NMR tube. All eight extracts were pooled, and the final solution was evaporated to dryness and re-dissolved in 1 mL of methanol-d₄. Then, 900 μL were collected and mixed with 50 μL of a 570 mM 1,3,5-trioxane solution (used as an internal quantification reference) and 1.5 mg of Cr(acac)_3_ for NMR analysis. The complete extraction was performed on the 12 injectable solutions in duplicate.

### 4.3. NMR Analysis

NMR experiments were conducted using a Bruker Avance 500 spectrometer (Bruker Biospin AG, Billerica, MA, USA) equipped with a 5 mm TCI cryoprobe (triple resonance inverse). Spectra were recorded at 298 K.

For qualitative ^1^H NMR analysis, the parameters used were as follows: an acquisition time of 2.04 s, a relaxation delay of 1.00 s, a spectral width of 8012 Hz (16 ppm), a time-domain size of 32,768 points, and a number of scans ranging from 16 (1 min) to 160 (8 min) to achieve a signal-to-noise ratio above 3. The 90° pulse length varied between 8.25 and 15.1 µs depending on the sample.

For quantitative ^1^H NMR analysis, the acquisition time was increased to 4.09 s, the relaxation delay was adjusted between 5.91 and 10.00 s, and the flip angle was reduced between 2.72 and 4.98 µs, corresponding to a magnetization flip angle of 30°. The number of scans varied from 64 to 500 to ensure a signal-to-noise ratio suitable for quantification (>10). For ^13^C NMR quantification, inverse gated experiments with ^1^H decoupling were recorded. The optimized parameters to achieve a signal-to-noise ratio around 30 were as follows: an acquisition time of 2.8 s, a relaxation delay of 13.5 s, and 872 scans collected with a flip angle of 30°. Under these conditions, the limit of quantification was estimated at 10 mM. A spectral width of 35,211 Hz (280 ppm) was used centered at 59 ppm. Longitudinal relaxation times (T_1_) were determined using an inversion-recovery pulse sequence with recovery delays ranging from 0.001 to 40 s for ^1^H or 60 s for ^13^C in 21 increments in both cases, ensuring a recovery rate above 97.5% for proton and 99.9% for carbon.

One-dimensional NMR data were then pre-processed using the Topspin 4.08 software (Bruker Biospin AG) with one level of zero-filling and Fourier transformation after multiplying FIDs by an exponential line-broadening function of 0.3 (^1^H) or 1 Hz (^13^C). Phase adjustment and polynomial baseline correction were carried out manually on each spectrum. All chemical shifts (δ) were referred to the ^1^H signal of TSP at 0 ppm or to the ^13^C signal of 1,3,5-trioxane at 96.3 ppm.

To achieve complete molecular assignment, a series of one- and two-dimensional experiments were conducted, including ^1^H-^1^H COSY, ^1^H-^13^C HSQC, ^1^H-^13^C HMBC, and ^13^C J-Mod. The parameters for these experiments were optimized accordingly, ensuring accurate structural elucidation of the studied molecules.

### 4.4. Chemometrics

Statistical analysis was conducted using SIMCA-P+ 13.0.3.0 (Umetrics, Umea, Sweden) and the open-source Rstudio^®^ (2024.09.0) software. The twenty-four ^13^C NMR spectra of the injectable solutions and the nine NMR spectra of the claimed active ingredients (trenbolone acetate, testosterone enanthate, testosterone cypionate, nandrolone decanoate, boldenone undecylenate, methenolone enanthate, testosterone propionate, drostanolone propionate, and trenbolone hexahydro-benzylcarbonate) were considered for statistical analysis. After spectra pre-processing described in Section 4.3, only the fingerprinting region of 0–50 ppm was considered for all samples. The data matrix was obtained by generating buckets for all NMR signals (55 in total) in the thirty-three spectra using the binning area method included in the KnowItAll^®^ software (Wiley Science Solutions, Hoboken, NJ, USA). For data pre-treatment, the normalization involved dividing each integrated region by the total spectral area. First, principal component analysis (PCA) was performed with mean-centered scaling on all injectable samples (24) to find any clustering between the formulations. Second, Pearson correlation coefficient was calculated on all spectra (33) using the *cor* function to identify anabolic steroids patterns. Third, a heatmap was generated on the twenty-four spectra of samples and the three NMR spectra of testosterone esters, using the ward.D2 method and the *hclustfun* function to compute the hierarchical clustering, in order to determine the presence of active ingredients.

### 4.5. Mass Spectrometry

The samples were dissolved in an acetonitrile–water mixture (CH_3_CN:H₂O 80:20 *v*/*v*) and analyzed using a Waters XEVO G2 mass spectrometer (Manchester, UK) in direct infusion mode. Injection was performed at a flow rate of 0.15 mL/min with a solvent mixture of acetonitrile and 0.1% aqueous formic acid in the same proportions as the dissolution medium. Ionization was achieved via electrospray ionization (ESI) in positive mode. High-resolution mass spectra (10^−4^ uma) were acquired using a quadrupole time-of-flight (Q-ToF) analyzer, with calibration performed using sodium formate, ensuring multi-point calibration between m/z 100 and 1200. Internal mass correction was applied in real time using leucine enkephalin ([M + H]⁺, *m*/*z* 556.2771).

Experimental parameters were set as follows: desolvation temperature at 350 °C, desolvation gas flow at 1100 L/h, cone voltage at 10 or 30 V, cone gas flow at 50 L/h to prevent aggregation, ion source temperature at 110 °C, and mass detection window between *m*/*z* 100 and 1500. For tandem mass spectrometry (MS/MS), the cone voltage was maintained at 30 V, and collision energies of 15, 25, and 35 V were applied, with a mass detection range from *m*/*z* 50 to 1000.

## Figures and Tables

**Table 1:** Formulations analyzed with claimed and detected AASs. Incorrect active ingredients are written in bold. Ostarine is actually a selective androgen receptor modulator (SARM) but the detected actives are AASs.

Sample Number	Form	Claimed Active Compound	Detected Active Compound
**1**	capsule	Androst-4-ene-3,11,17-trione	Androst-4-ene-3,11,17-trione
**2**	tablet	Methandienone	Methandienone
**3**	capsule	Epistane	Epistane
**4**	tablet	Oxandrolone	**Stanozolol**
**5**	tablet	Methyl-1-testosterone	Methyl-1-testosterone
**6**	tablet	Methandienone	Methandienone
**7**	capsule	Ostarine	**Testosterone decanoate Stanozolol**
**8**	tablet	Oxandrolone	Oxandrolone
**9**	tablet	Oxymetholone	**Methyltestosterone**
**10**	tablet	Mesterolone	Mesterolone
**11**	tablet	Stanozolol	Stanozolol
**12**	tablet	no information *	Stanozolol
**13**	tablet	no information *	Methandienone
**14**	tablet	no information *	Stanozolol
**15**	injectable	Boldenone undecylenate	Boldenone undecylenate
**16**	injectable	Boldenone undecylenate	Boldenone undecylenate
**17**	injectable	Methenolone enanthate	Methenolone enanthate
**18**	injectable	Testosterone cypionate	Testosterone cypionate
**19**	injectable	Testosterone propionate	Testosterone propionate
**20**	injectable	Trenbolone enanthate	Trenbolone enanthate

* These tablets were seized in white boxes without any labeling.

**Table 2:** Characteristic signals for ^1^H NMR quantification of AASs. Spectra were recorded in methanol-d4.

Active Compound	Detected in Sample	Signal(s) Used for Quantification	
		**δ (ppm), Multiplicity ^a^, J (Hz),** **Number of Protons**	**Assignment ^b^**
Androst-4-ene-3,11,17-trione	**1**	5.76 ppm, s, 1H	H4
Methandienone	**2**, **6**, **13**	7.31, d (10.0), 1H	H1
		6.21, dd (10.0, 1.9), 1H	H2
		6.06 apt (1.9), 1H	H4
Epistane	**3**	3.11, m, 1H	H4/H6
Stanozolol	**4**, **7**, **11**, **12**, **14**	7.24, s, 1H	H5
		0.73, s, 3H	H24
Methyl-1-testosterone	**5**	7.31, d (10.2), 1H	H1
		5.83 dd (10.2, 0.7), 1H	H2
Testosterone decanoate	**7**	5.70, s, 1H	H4
Oxandrolone	**8**	AB system, 4.26 (part A) and 4.01	
		(part B) (10.7), 2H	H3
		1.83, s, 3H	H18
Methyltestosterone	**9**	5.71, s, 3H	H4
Mesterolone	**10**	2.79, dd (14.7, 6.0), 1H	H2
Boldenone undecylenate	**15**, **16**	6.20, dd (10.1, 1.9), 1H	H2
		6.05, apt (1.5), 1H	H4
Methenolone enanthate	**17**	5.71, s, 1H	H2
Testosterone cypionate	**18**	5.70, s, 1H	H4
Testosterone propionate	**19**	5.72, s, 1H	H4
Trenbolone enanthate	**20**	AB system, 6.55 (part A) and 6.40	
		(part B) (9.9), 2H	H11, H12
		5.77, s, 1H	H4

^a^ s singlet, d doublet, dd doublet of doublet, apt apparent triplet, m multiplet. ^b^ See numbering in Figure A1 (Appendix A.1).

**Table 3:** Oil-based injectable formulations with claimed and detected AASs and their dosage.

Oil-Based Injectable Samples	Claimed Active Compound	Claimed Content (mg/mL)	Detected Active Compound (Testosterone Esters)	Amount mg/mL (% Found/Claimed)
**OI-1**	Trenbolone acetate	120	propionate	56 ± 1
**OI-2**	Testosterone enanthate	150	enanthate cypionate	25 ± 1 (17%) 25 ± 1 (17%)
	Testosterone cypionate	150		
	Nandrolone decanoate	150		
**OI-3**	Boldenone undecylenate	250	cypionate	65 ± 2
			propionate	42 ± 3
**OI-4**	Testosterone cypionate	300	cypionate	61 ± 3 (20%)
			propionate	41 ± 2
**OI-5**	Nandrolone decanoate	300	Enanthate	58 ± 2
			cypionate	50 ± 2
**OI-6**	Methenolone	120	cypionate	69 ± 4
			propionate	41 ± 2
**OI-7**	Testosterone propionate	120	propionate	41 ± 2 (34%)
			cypionate	62 ± 3
**OI-8**	Testosterone propionate	100	propionate	49 ± 1 (49%)
	Trenbolone acetate	100		
	Drostanolone propionate	100		
**OI-9**	Testosterone propionate	60	propionate cypionate	44 ± 2 (73%) 71 ± 1 (59%)
	Testosterone enanthate	120		
	Testosterone cypionate	120		
**OI-10**	Testosterone enanthate	300	cypionate	69 ± 4
			propionate	47 ± 3
**OI-11**	Testosterone enanthate	75	propionate	34 ± 2
	Trenbolone acetate	75		
	Trenbolone hexahydrobenzyl carbonate	75		
**OI-12**	Testosterone enanthate	75	propionate	48 ± 1
	Trenbolone acetate	75		
	Trenbolone hexahydrobenzyl carbonate	75		

**Figure A1:**
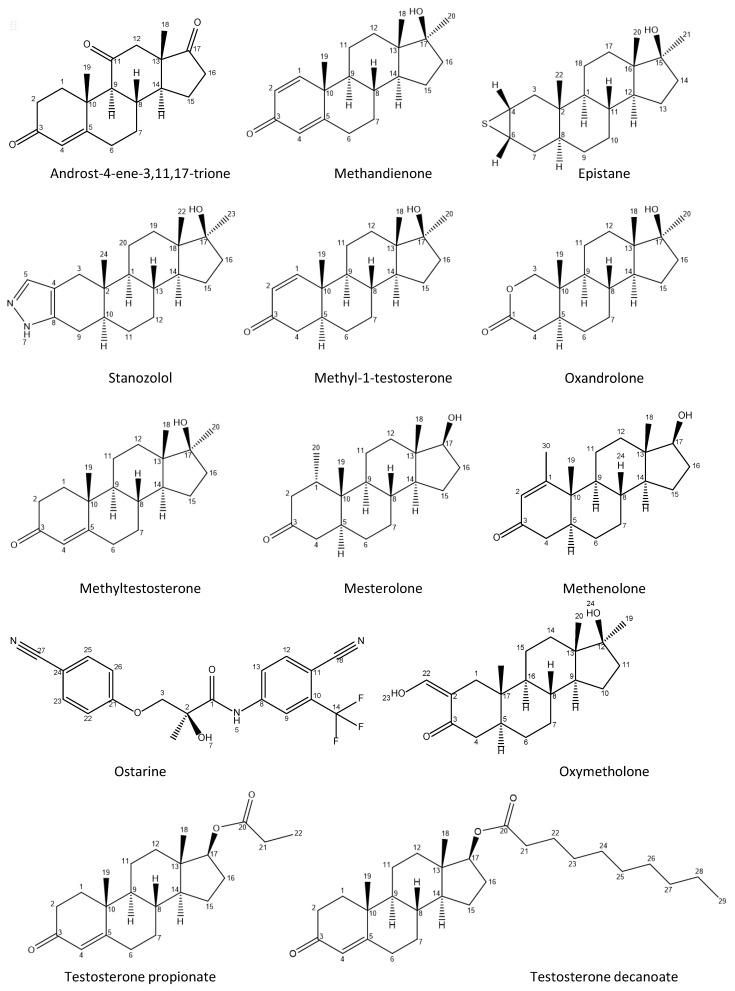

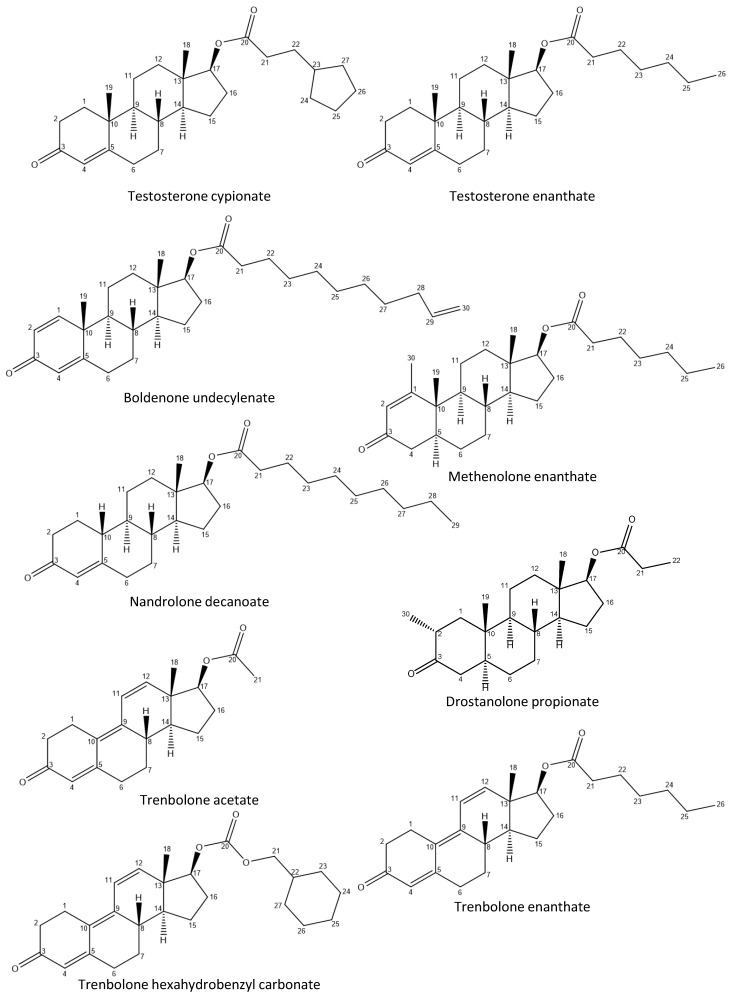
Numbered structures of the AAS claimed and/or detected in the formulations analyzed.

## Data Availability

Data are contained within the article and Supplementary Materials.

## Supplementary Materials

The following supporting information can be downloaded at https://www.mdpi.com/article/10.3390/molecules30092060/s1; Table S1: Accurate mass data of active compounds detected in the 20 AAS formulations analyzed [25]; Table S2: Formulations analyzed with claimed and detected AAS and their dosage; Table S3: Accurate mass data of active compounds detected in the 12 oil-based injectable formulations analyzed; Figure S1: Loading plot of the PCA built from the twelve oil-based injectable formulations analyzed in duplicate (*n* = 24); Figure S2: Overlay of HSQC spectra of the three testosterone esters in the 0–50 ppm carbon region.

## Abbreviations

The following abbreviations are used in this manuscript:

NMR	Nuclear magnetic resonance
AAS	Anabolic androgenic steroids
TSP	Sodium 2,2,3,3-tetradeutero-3-trimethylsilyl-propionate
MS	Mass spectrometry
PCA	Principal component analysis
HCA	Hierarchical cluster analysis

## Appendix A

### Appendix A.2

**Table A1:** ^1^H and ^13^C assignments of the three standard testosterone esters. Spectra were recorded in methanol-d4. Signals used for the quantification are in bold.

	Testosterone Cypionate		Testosterone Propionate		Testosterone Enanthate	

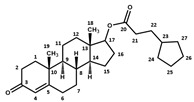
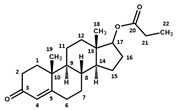
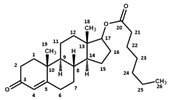
			
Atom N°	δ ^1^H (ppm)	δ ^13^C (ppm)	δ ^1^H (ppm)	δ ^13^C (ppm)	δ ^1^H (ppm)	δ ^13^C (ppm)
1	2.08; 1.69	38.4	2.09; 1.69	38.5	2.07; 1.69	38.8
2	2.48; 2.31	35.5	2.49; 2.28	35.6	2.475; 2.28	35.9
3	-	204.0	-	204.0	-	204.2
4	5.71	125.9	5.705	126.0	5.70	126.3
5	-	176.6	-	176.6	-	176.8
6	2.31; 2.48	36.4	2.31; 2.49	36.4	2.31; 2.475	36.7
7	1.06; 1.91	34.5	1.06; 1.91	34.5	1.05; 1.90	34.8
8	1.70	38.3	1.70	38.4	1.70	38.6
9	1.01	56.9	1.01	57.0	1.00	57.3
10	-	41.7	-	41.7	-	42.0
11	1.48; 1.61	23.4	1.48; 1.61	23.4	1.47; 1.61	23.7
12	1.22; 1.79	39.6	1.22; 1.79	39.7	1.21; 1.78	40.0
13	-	45.4	-	45.5	-	45.8
14	1.12	53.2	1.12	53.3	1.11	53.6
15	1.70; 1.43	26.1	1.70; 1.41	26.1	1.69; 1.41	26.4
16	2.17; 1.54	30.2	2.17; 1.54	30.3	2.15; 1.53	30.6
17	4.61	85.6	4.61	85.6	4.61	85.8
18	0.89	14.2	0.88	14.2	0.88	14.5
19	1.25	19.4	1.24	19.4	1.24	19.7
20	-	177.4		178.0	-	177.5
21	2.33	36.4	2.33	30.3	2.31	37.3
22	1.62	34.2	1.11	**11.3**	1.60	28.2
23	1.79	**42.6**			1.31	34.7; 31.9; 25.6
24	1.79; 1.12	35.1			1.31	34.7; 31.9; 25.6
25	1.71; 1.54	27.8			1.31	34.7; 31.9; 25.6
26	1.71; 1.54	27.8			0.90	**16.4**
27	1.79; 1.12	35.1				
