# Supplementary material for: Quantitative 1H and 13C NMR and Chemometric Assessment of 13C NMR Data: Application to Anabolic Steroid Formulations
Source: Molecules. 2025 May 6;30(9):2060. doi: 10.3390/molecules30092060 (PMC12074264; doi:10.3390/molecules30092060)

Table S1. Accurate mass data of active compounds detected in the 20 AAS formulations analyzed. Incorrect active ingredients are written in bold

Sample number	Detected active compound	$[M+H]^+$ formula	Measured accurate m/z value	mass error (ppm)	m/z of major characteristic fragment ions
1	androst-4-ene-3,11,17-trione	$[C_{19}H_{25}O_3]^+$	301.1803	-0.3	109.0656<br>121.0655<br>257.1550
2	Methandienone	$[C_{20}H_{29}O_2]^+$	301.2165	-1.0	121.0655
3	Epistane	$[C_{20}H_{33}OS]^+$	303.2148 (-H_2O)*	+0.7	269.2270
4	**Stanozolol**	$[C_{21}H_{33}N_2O]^+$	329.2592	-0.3	81.0456<br>121.1017
5	Methyl-1-testosterone	$[C_{20}H_{31}O_2]^+$	303.2325	+0.3	201.1653<br>267.2115<br>285.2222
6	Methandienone	$[C_{20}H_{29}O_2]^+$	301.2171	+1.0	121.0653<br>149.1334<br>283.2067
7	**Testosterone decanoate**	$[C_{29}H_{47}O_3]^+$	443.3511	+3.2	97.0649<br>109.0644<br>155.1429<br>253.1947<br>271.2047
	**Stanozolol**	$[C_{21}H_{33}N_2O]^+$	329.2585	-2.4	81.0452<br>121.1011
8	Oxandrolone	$[C_{19}H_{31}O_3]^+$	307.2272	-0.3	271.2059<br>289.2164
9	**Methyltestosterone**	$[C_{20}H_{31}O_2]^+$	303.2328	+1.3	97.0653<br>109.0652<br>285.2214
10	Mesterolone	$[C_{20}H_{33}O_2]^+$	305.2479	-0.7	229.1970<br>269.2262<br>287.2386
11	Stanozolol	$[C_{21}H_{33}N_2O]^+$	329.2598	+1.5	81.0456<br>121.1016
12	Stanozolol	$[C_{21}H_{33}N_2O]^+$	329.2595	+0.6	81.0452<br>121.1014
13	Methandienone	$[C_{20}H_{29}O_2]^+$	301.2167	-0.3	121.0649<br>149.1326<br>283.2058
14	Stanozolol	$[C_{21}H_{33}N_2O]^+$	329.2595	+0.6	81.0452<br>121.1014
15	Boldenone undecylenate	$[C_{30}H_{45}O_3]^+$	453.3364	-1.1	121.0652<br>135.1174<br>269.1908
16	Boldenone undecylenate	$[C_{30}H_{45}O_3]^+$	453.3376	+1.6	121.0658<br>135.1179<br>269.1911

17	Methenolone enanthate	$[C_{27}H_{43}O_3]^+$	415.3212	0.0	113.0966<br>187.1486<br>303.2314
18	Testosterone cypionate	$[C_{27}H_{41}O_3]^+$	413.3054	-0.5	97.0658<br>253.1961<br>271.2065
19	Testosterone propionate	$[C_{22}H_{33}O_3]^+$	345.2433	+0.9	97.0651<br>109.0650
20	Trenbolone enanthate	$[C_{25}H_{35}O_3]^+$	383.2592	+1.6	253.1598

* $[M+H]^+$ ion was not detected, the most abundant ion was neutral loss of water at m/z 303 as previously described (Okano, M.; Sato, M.; Ikekita, A. Analysis of non-ketoic steroids 17α-methylepithiostanol and desoxymethyl-testosterone indietary supplements. Drug Test. Anal. 2009, 1, 518–525).

Table S2. Formulations analyzed with claimed and detected AAS and their dosage. Incorrect active ingredients or dosages are written in bold. Ostarine is actually a selective androgen receptor modulator (SARM) but the detected actives are AAS.

Sample number	Form	Claimed active compound	Claimed content (mg/ml)	Detected active compound	Amount mg/unit[b] (%found/claimed)	Compliance Active/Dosage
1	capsule	Androst-4-ene-3,11,17-trione	100	Androst-4-ene-3,11,17-trione	**80.4 ± 3.8** (80 ± 4%)	Yes/No
2	tablet	Methandienone	10	Methandienone	9.6 ± 0.4 (96 ± 4%)	Yes//Limit
3	capsule	Epistane	20	Epistane	19.9 ± 0.1 (100 ± 1%)	Yes/Yes
4	tablet	Oxandrolone	**10**	**Stanozolol**	4.5 ± 0.1	No
5	tablet	Methyl-1-testosterone	10	Methyl-1-testosterone	10.1 ± 0.8 (101 ± 8%)	Yes/Yes
6	tablet	Methandienone	10	Methandienone	10.1 ± 0.8 (101 ± 8%)	Yes/Yes
7	capsule	Ostarine	**6.5**	**Testosterone decanoate**<br>**Stanozolol**	5.5 ± 0.3<br>1.2 ± 0.1	No
8	tablet	Oxandrolone	10	Oxandrolone	**8.8 ± 0.1** (88 ± 1%)	Yes/No
9	tablet	Oxymetholone	**25**	**Methyltestosterone**	2.3 ± 0.1	No
10	tablet	Mesterolone	25	Mesterolone	24.3 ± 0.1 (97 ± 1%)	Yes/Yes
11	tablet	Stanozolol	10	Stanozolol	9.5 ± 0.3 (95 ± 3%)	Yes/Limit
12	tablet	no information [a]		Stanozolol	4.9 ± 0.2	
13	tablet	no information [a]		Methandienone	4.7 ± 0.2	
14	tablet	no information [a]		Stanozolol	4.3 ± 0.1	
15	injectable	Boldenone undecylenate	250	Boldenone undecylenate	**215 ± 6** (86 ± 3%)	Yes/No
16	injectable	Boldenone undecylenate	200	Boldenone undecylenate	**174 ± 2** (87 ± 1 %)	Yes/No
17	injectable	Methenolone enanthate	100	Methenolone enanthate	96 ± 2 (96 ± 2%)	Yes/ Limit
18	injectable	Testosterone cypionate	250	Testosterone cypionate	239 ± 4 (96 ± 2%)	Yes/ Limit
19	injectable	Testosterone propionate	100	Testosterone propionate	**87 ± 3** (87 ± 3%)	Yes/No
20	injectable	Trenbolone enanthate	200	Trenbolone enanthate	192 ± 3 (96 ± 2%)	Yes/Limit

[a] These tablets were seized in white boxes without any labeling.

[b] For injectable formulations containing 10 mL of solution, the injected dose (unit) is considered to be 1 mL.

Table S3. Accurate mass data of active compounds detected in the 12 oil-based injectable formulations analyzed.

Sample number	Detected active compound	[M+H]+ formula	Measured accurate m/z value	mass error (ppm)	m/z of major characteristic fragment ions
OI-1	Testosterone propionate	$[C_{22}H_{33}O_3]^+$	345.2422	-2.3	97.0661; 109.0660; 253.1963; 271.2066
OI-2	Testosterone enanthate	$[C_{26}H_{41}O_3]^+$	401.3058	0.5	97.0653; 109.0652; 253.1954; 271.2061
	Testosterone cypionate	$[C_{27}H_{41}O_3]^+$	413.3059	0.7	97.0650; 109.0650; 175.1485; 253.1956; 271.2058
OI-3	Testosterone cypionate	$[C_{27}H_{41}O_3]^+$	413.3054	-0.5	97.0653; 109.0651; 175.1488; 253.1956; 271.2067
	Testosterone propionate	$[C_{22}H_{33}O_3]^+$	345.2428	-0.6	97.0650; 109.0650; 253.1956; 271.2063
OI-4	Testosterone cypionate	$[C_{27}H_{41}O_3]^+$	413.3062	1.5	97.0654; 109.0651; 253.1960
	Testosterone propionate	$[C_{22}H_{33}O_3]^+$	345.2433	0.9	97.0652; 109.0651; 253.1958; 271.2062
OI-5	Testosterone enanthate	$[C_{26}H_{41}O_3]^+$	401.3057	0.2	97.0653; 109.0652; 175.1487; 253.1961
	Testosterone cypionate	$[C_{27}H_{41}O_3]^+$	413.3058	0.5	97.0650; 109.0652; 253.1956; 271.2058
OI-6	Testosterone cypionate	$[C_{27}H_{41}O_3]^+$	413.3051	-1.2	97.0653; 109.0653; 253.1959
	Testosterone propionate	$[C_{22}H_{33}O_3]^+$	345.2425	-1.4	97.0659; 109.0658; 253.1958; 271.2064
OI-7	Testosterone cypionate	$[C_{27}H_{41}O_3]^+$	413.3057	0.2	97.0654; 109.0653; 253.1956; 175.1486; 271.2058
	Testosterone propionate	$[C_{22}H_{33}O_3]^+$	345.2430	0	97.0651; 109.0649; 253.1953; 271.2058
OI-8	Testosterone propionate	$[C_{22}H_{33}O_3]^+$	345.2424	-1.7	97.0657; 109.0656; 175.1489; 253.1959
OI-9	Testosterone cypionate	$[C_{27}H_{41}O_3]^+$	413.3057	0.2	97.0656; 109.0656; 175.1489; 253.1956
	Testosterone propionate	$[C_{22}H_{33}O_3]^+$	345.2430	0	97.0654; 109.0653; 253.2065; 271.2065
OI-10	Testosterone cypionate	$[C_{27}H_{41}O_3]^+$	413.3056	0	97.0651; 109.0650; 175.1486; 253.1956
	Testosterone propionate	$[C_{22}H_{33}O_3]^+$	345.2429	-0.3	97.0659; 109.0658; 253.1960; 271.2065
OI-11	Testosterone propionate	$[C_{22}H_{33}O_3]^+$	345.2428	-0.6	97.0654; 109.0652; 253.1956; 271.2062
	Testosterone cypionate*	$[C_{27}H_{41}O_3]^+$	413.3050	-1.4	97.0654; 109.0650; 253.1956
	Trenbolone acetate*	$[C_{20}H_{25}O_3]^+$	313.1801	-1.0	253.1594; 271.1699
OI-12	Testosterone propionate	$[C_{22}H_{33}O_3]^+$	345.2431	0.3	97.0650; 109.0649; 253.1958; 271.2065
	Trenbolone acetate*	$[C_{20}H_{25}O_3]^+$	313.1805	0.3	253.1595; 271.1700

* These compounds were detected as minor signals in the MS spectra.

Figure S1: Loading plot of the PCA built from the twelve oil-based injectable formulations analyzed in duplicate (n=24). The variables correspond to ^{13}C NMR signals. Values are chemical shift in ppm.

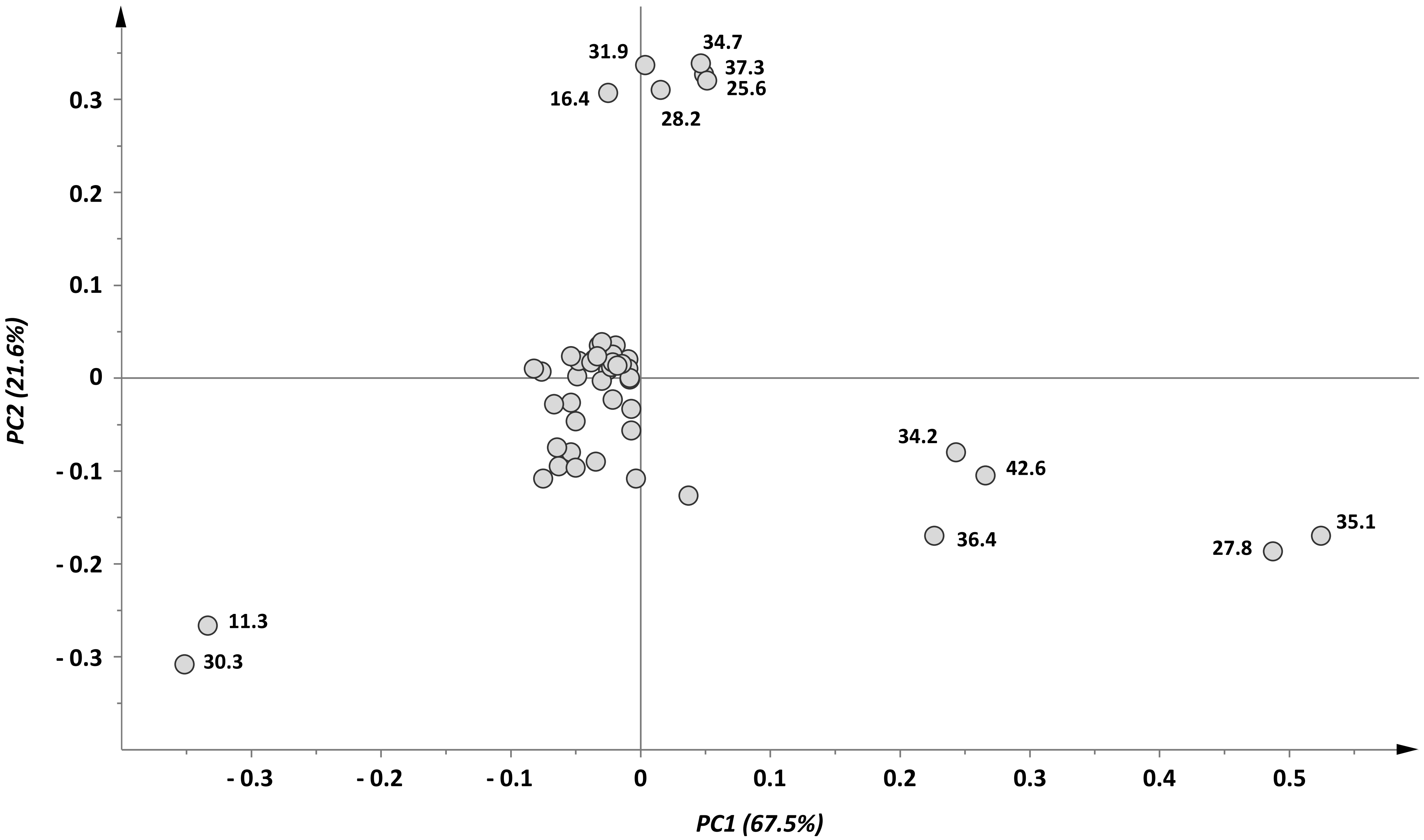

Figure S2: Overlay of HSQC spectra of the three testosterone esters in the 0–50 ppm carbon region. The colored signals correspond to specific resonances in the ester moieties: testosterone cypionate (blue), testosterone propionate (red), and testosterone enanthate (green). The black signals indicate non-specific resonances that overlap across the different ester structures. Spectra were recorded at 500 MHz in methanol-d_4.

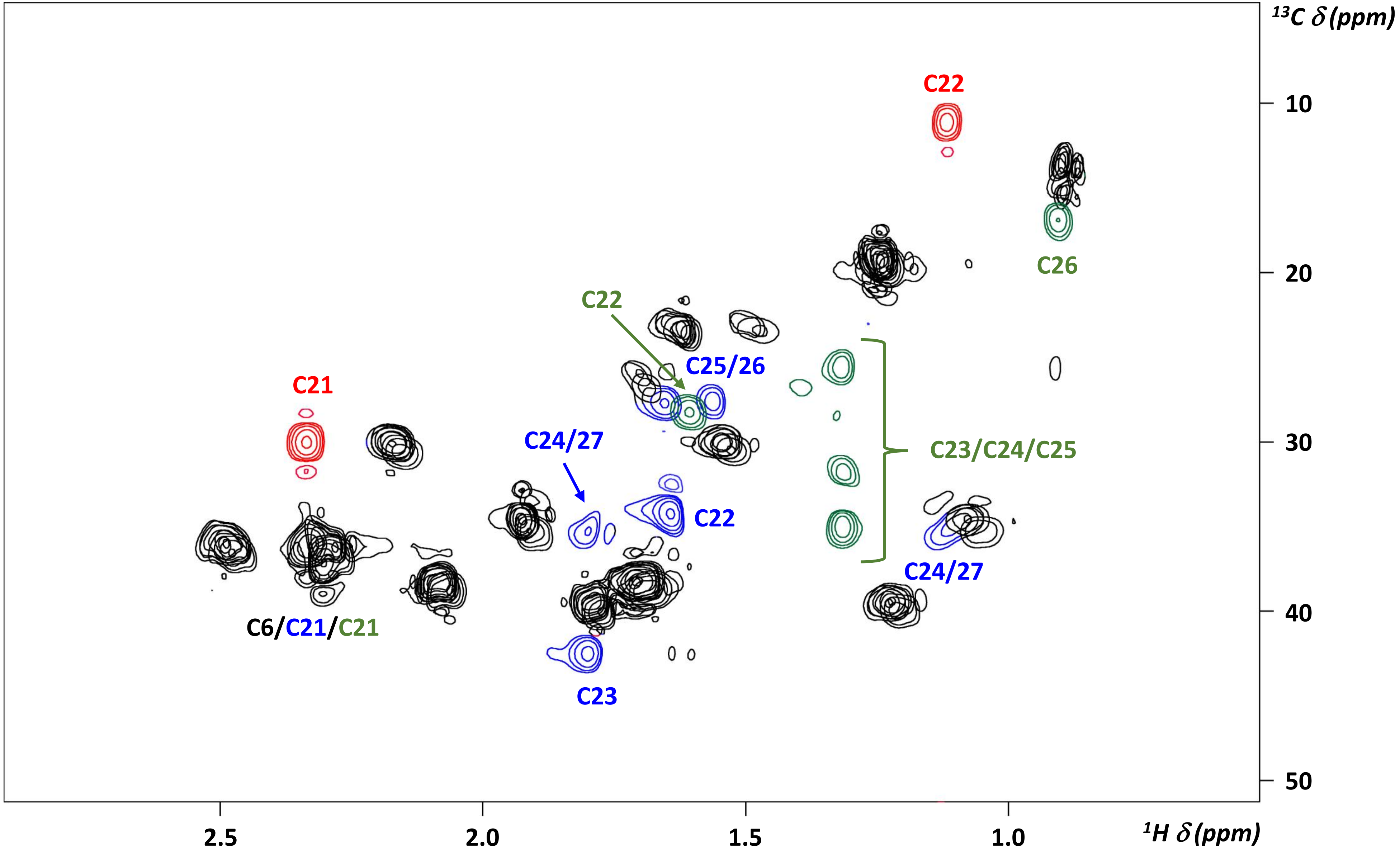

Supplement: Supplementary file 1 [file molecules-30-02060-s001.zip › molecules-3624214-supplementary.pdf]
